# Expositionstherapie bei Panikstörung und Agoraphobie im Kontext bestehender antidepressiver Medikation

**DOI:** 10.1007/s00115-023-01535-y

**Published:** 2023-08-29

**Authors:** Malte Hahnfeld, Philipp Ritter, Cathrin Sauer, Kerstin Weidner, René Noack

**Affiliations:** 1 Klinik und Poliklinik für Psychotherapie und Psychosomatik, Universitätsklinikum Carl Gustav Carus Dresden, Technische Universität Dresden, Fetscherstr. 74, 01307 Dresden, Deutschland; 2 Klinik und Poliklinik für Psychiatrie und Psychotherapie, Universitätsklinikum Carl Gustav Carus Dresden, Technische Universität Dresden, Fetscherstr. 74, 01307 Dresden, Deutschland

**Keywords:** Kognitive Verhaltenstherapie, Antidepressiva, Kombinationsbehandlung, Angststörungen, Tagesklinik, Cognitive behavioral therapy, Antidepressants, Adjunction, Anxiety disorders, Day hospital

## Abstract

**Hintergrund:**

Kognitive Verhaltenstherapie (KVT) und Pharmakotherapie mit Antidepressiva stellen bei Agoraphobie und/oder Panikstörung jeweils hochwirksame Behandlungsmethoden dar. Eine Kombination von KVT und Antidepressiva wird jedoch aufgrund potenziell ungünstiger Interferenzeffekte diskutiert. Untersucht wurden die Assoziationen einer bestehenden antidepressiven Medikation mit Panik- und agoraphobischer Symptombelastung und deren Veränderung im Kontext einer strukturierten fünfwöchigen tagesklinischen und auf Expositionen fokussierten Behandlung in einem naturalistischen Setting.

**Methodik:**

Von insgesamt *n* = 488 Patient*innen konnte für *n* = 380 retrospektiv die Medikamenteneinnahme während der Behandlung ermittelt werden: *n* = 100 (26,3 %) nahmen Antidepressiva verschiedener Wirkstoffklassen ein. Die Berechnungen erfolgten mittels multipler linearer Regressionsanalyse, t‑Tests, Responseanalysen und χ^2^-Tests.

**Ergebnisse:**

Patient*innen mit bestehender antidepressiver Medikation erfüllten häufiger die Kriterien einer komorbiden depressiven Störung (*p* < 0,001). Das Maß der Symptomveränderung und die Therapieresponseraten unterschieden sich nicht zwischen Patient*innen mit und ohne Antidepressiva in Bezug auf die Angstsymptomatik.

**Diskussion:**

Im untersuchten Kontext profitierten Patient*innen mit und ohne bestehende antidepressive Medikation in gleichem Maße von KVT in Bezug auf die Angstsymptomatik.

## Abstract (en)

**Background:**

Cognitive behavioral therapy (CBT) and pharmacotherapy with antidepressants are both a highly effective treatment for agoraphobia and/or panic disorder; however, a combination of CBT and antidepressants is under debate due to potentially unfavorable interference effects. The associations of existing antidepressant medication with panic and agoraphobia symptom burden and their change in the context of a structured 5‑week day hospital and exposure-focused treatment in a naturalistic setting were investigated.

**Methods:**

Out of a total of *n* = 488 patients medication use during treatment was retrospectively determined for *n* = 380: *n* = 100 (26.3%) were taking antidepressants of different drug classes. Calculations were performed using multiple linear regression analysis, t‑tests, response analyses, and χ^2^-tests.

**Results:**

Patients with existing antidepressant medication more often met the criteria for comorbid depressive disorder (*p* < 0.001). The measure of symptom change and treatment response rates did not differ between patients with and without antidepressants with respect to anxiety symptoms.

**Discussion:**

In the context studied, patients with and without existing antidepressant medication benefited equally from CBT with respect to anxiety symptoms.

## Theoretischer Hintergrund

Agoraphobie mit oder ohne Panikstörung und die isolierte Panikstörung sind häufig [12] und beeinträchtigen Lebensqualität und berufliche Teilhabe stark [18, 19]. Betroffene zeigen eine hohe Inanspruchnahme medizinischer Leistungen [13]. Der Verlauf ist oft chronisch rezidivierend [5, 29], Spontanremissionen sind selten [27].

Die S3-Leitlinien zur Behandlung von Angststörungen ordnen der kognitiven Verhaltenstherapie (KVT) und der psychopharmakologischen Behandlung mit Antidepressiva (AD) jeweils den höchsten Empfehlungsgrad zu [4]. Hier findet sich auch die Empfehlung einer Kombination unter Berücksichtigung des Schweregrads der Symptomatik, der Funktionsbeeinträchtigung und der Präferenz der Patient*innen. Bei Nichtwirken der einen oder der anderen singulär angewandten Behandlung soll nach Expert*innenkonsens in den Leitlinien die jeweils andere oder eine Kombinationsbehandlung stattfinden. Jedoch ist das Ausmaß der Überlegenheit der Kombination gegenüber Monotherapie, z. B. KVT allein, nicht nur in Bezug auf Langzeiteffekte, sondern auch hinsichtlich der Effektivität in der Akutbehandlungsphase wider Erwarten gering [22]. Anwendungsrealität ist jedoch, dass ein großer Teil der Patient*innen, die eine KVT aufnehmen, bereits AD einnehmen. Bei Nakano et al. [20] waren es in einem ambulanten Setting ca. 60 % der Patient*innen mit Panikstörung.

Ziel der hier beschriebenen Studie ist es, in einem naturalistischen Setting mit großer Stichprobe an Patient*innen, welche alle dieselbe hohe Intensität an KVT erhielten, die Unterschiede in der Wirksamkeit dieser KVT in Abhängigkeit von einer bereits vorliegenden Medikation mit AD zu untersuchen. Anzunehmen wäre eine geringere Wirksamkeit von KVT bei Patient*innen mit AD vor dem Hintergrund, dass die S3-Leitlinien in ihrer zusammenfassenden Sicht auf die Studienlage eine in vielen Arbeiten kaum sichtbare Überlegenheit einer Kombinationsbehandlung gegenüber einer rein medikamentösen Behandlung beschreiben. Im hier vorliegenden Design, lässt sich das vorherige Wirksamkeitsausmaß der vorher verabreichten AD auf die Angstsymptomatik nicht mehr quantifizieren. Insofern wird die Diskussion der Vergleiche eingeschränkt sein, im Sinne dass, wie Lee et al. [17] postulieren, möglicherweise ein großer Anteil Nonresponder auf AD hier auf die andere Therapieoption KVT ansprechen könnten, während vorherige Responder auf AD in der Stichprobe fehlen könnten. Anzunehmen wäre jedoch ein zumindest teilweise bereits vorher erfolgter positiver Effekt durch das AD auf die Angstsymptomatik, welcher sich durch eine zusätzliche KVT also nur noch wenig steigern ließe und den antidepressiv nicht Vorbehandelte nicht erlebten.

Zur Wirksamkeit von KVT unter dem Einfluss von AD gibt es bisher keine Evidenz, dass AD Lernmechanismen im Kontext einer KVT fördern [17]. Umgekehrt können jedoch neurobiologische Mechanismen herangezogen werden, die eine Beeinträchtigung psychotherapeutischer Lernprozesse erklären könnten. Zum einen postulieren Otto et al. [22], die Gabe von AD hemme die Ausschüttung von Glukokortikoiden. Eine geringere Glukokortikoidaktivität korreliere demnach mit einer reduzierten Gedächtniskonsolidierung und könnte damit das Extinktionslernen der Patient*innen stören. Burghardt et al. [7] stellten zum anderen die Theorie auf, dass die chronische Einnahme von AD amygdalaabhängiges Lernen störe. Im Tiermodell führte die Behandlung mit Citalopram über 22 Tage zu beeinträchtigtem Extinktionslernen.

Zudem könnten ungünstige Attributionseffekte durch die Zuschreibung des Behandlungsfortschritts auf die Medikation die Motivation für KVT und so den Erfolg einer Kombinationstherapie beeinträchtigen [1].

Zwei vorherige naturalistische Studien kamen zu dem Ergebnis, dass eine bestehende antidepressive Medikation keine signifikante Vorhersagekraft für das Ergebnis einer KVT bei der Behandlung hat [20, 25]. Der Anteil derjenigen Patient*innen mit antidepressiver Medikation war jedoch sehr gering (*n* = 34 und *n* = 17). Arch und Craske [2] fanden dagegen bei einer etwas größeren Stichprobe (*n* = 17 mit SSRI; *n* = 48 mit anderen AD) schlechtere Outcomes von KVT bei Patient*innen mit einer AD-Medikation zu Behandlungsbeginn.

## Methoden

### Design der Studie

**Figure 1:**
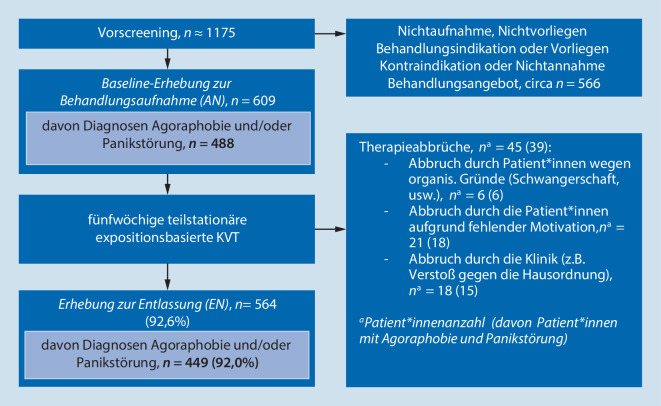

Es wird eine retrospektive Kohortenstudie präsentiert. Es wurden Unterschiede in den Parametern Symptomatik zu Beginn und Therapieende zwischen den Gruppen der Patient*innen mit bzw. ohne AD untersucht. Alle Patient*innen durchliefen ein stets fünfwöchiges tagesklinisches Behandlungsprogramm für Angst- und Zwangsstörungen in geschlossenen Gruppen mit 6 bis 9 Patient*innen, orientiert am Therapiemanual von Lang et al. [16], ohne parallele andere Behandlung. Neben den täglichen Gruppenpsychotherapien fanden wöchentlich 4 zeitoffene, therapeutisch begleitete Einzelexpositionssitzungen statt. Eine ausführliche Beschreibung des Behandlungskonzepts findet sich bei Beiling et al. [6] und Noack et al. [21]. Daten zur Symptombelastung und zur AD-Einnahme wurden zu Behandlungsbeginn (AN) und zu Behandlungsende (EN) erhoben – siehe Instrumente. Für die wissenschaftliche Verwendung der Daten gaben die Patient*innen ihre informierte Einwilligung. Das Patient*innenflussdiagramm ist in Abb. 1 dargestellt.

### Stichprobe

In die Studie wurden alle 488 Patient*innen mit den Diagnosen Agoraphobie und/oder Panikstörung sowie isolierter Panikstörung der insgesamt 609 Patient*innen einbezogen, die sich im Zeitraum zwischen Januar 2009 und Februar 2020 in Behandlung befanden. Die Aufnahme zur Behandlung erfolgte konsekutiv. Im Zuge eines vorherigen ambulanten Vorscreenings wurden Behandlungsindikationen und Kontraindikationen durch die klinische Einschätzung von mindestens zwei approbierten Psychotherapeut*innen und mindestens einer/einem Ärztin/Arzt überprüft. Im gesamten Erhebungszeitraum wurden retrospektiv geschätzt etwa 1175 Patient*innen voruntersucht, bei 2 bis 3 Screenings pro Woche und 42 Arbeitswochen im Jahr. Indikationen für eine Behandlung waren das Vorliegen einer Angst- oder Zwangserkrankung, Volljährigkeit, ausreichende Behandlungsmotivation sowie das Vorliegen eines nichtsuffizienten ambulanten Psychotherapieversuchs bzw. deren Nichterreichbarkeit. Kontraindikationen waren das Vorliegen einer psychotischen Erkrankung, Dissoziation oder Intrusion sowie somatische Faktoren, welche eine Konfrontationstherapie ausschließen. Beim Vorliegen von Abhängigkeitserkrankungen wurde vor Behandlungsbeginn eine längerfristige Abstinenz geprüft.

Von den *n* = 488 Patient*innen waren 64,8 % weiblich. Das durchschnittliche Alter war 37,2 Jahre (SD 12,8). Beim höchsten Schulabschluss hatten 82,0 % mindestens mittlere Reife, 72,3 % hatten bereits mindestens eine ambulante oder stationäre Vorbehandlung. Tab. 1 zeigt Diagnosen und Diagnosenanzahl. Die Vergabe klinischer Diagnosen erfolgte auf Basis des strukturierten klinischen Interviews für DSM(Diagnostic and Statistical Manual of Mental Disorders)-IV, Achse I und II (SKID I und II; [28]) durch trainierte Interviewer*innen.

**Table 1:** 

Diagnosen		Total	
		*n*	%
Hauptdiagnose	Agoraphobie mit Panikstörung	371	76,0
	Agoraphobie ohne Panikstörung	24	4,9
	Panikstörung ohne Agoraphobie	93	19,1
Komorbide Störungen	Andere Angststörungen und Zwangsstörungen	179	36,7
	Affektive Störungen	213	43,6
	Substanzbezogene Störungen	34	7,0
	Somatoforme Störungen	39	8,0
	Posttraumatische Belastungsstörung	23	4,7
	Persönlichkeitsstörungen	12	2,5
	Essstörungen	7	1,4
Anzahl Diagnosen	*MW (SD)*	*2,06*	*(1,00)*
	1	158	32,4
	2	195	40,0
	3	100	20,5
	4	35	7,2

*M* Mittelwert, *SD* Standardabweichung

### Medikation

Von der Gesamtstichprobe von *n* = 488 ließen sich für *n* = 380 die antidepressive Medikation retrospektiv aus den Akten nachvollziehen. Von diesen nahmen *n* = 134 Patient*innen (35,3 %) der Untersuchungsstichprobe während der Therapie mindestens ein Antidepressivum ein, davon *n* = 13 (9,7 %) zwei verschiedene Präparate. Bei einem Großteil der Patient*innen, *n* = 79 (59,0 %), blieb die Dosis bis zum Therapieende unverändert. Während des Behandlungszeitraumes erhöhten *n* = 5 (3,7 %) die Dosis, *n* = 11 (8,2 %) reduzierten die Dosis und bei *n* = 5 (3,7 %) wurde das Präparat durch ein anderes ersetzt. Bei *n* = 7 (5,2 %) wurde während der Therapie eine neue Medikation angesetzt und *n* = 27 (20,1 %) setzten das Präparat während der Behandlung ab. Die beiden zuletzt genannten Verlaufskategorien (Medikation an- und abgesetzt) wurden aus der statistischen Auswertung ausgeschlossen.

Eine detaillierte Übersicht über eingenommene Präparate und Dosis der *n* = 100 Patient*innen, welche in die statistische Auswertung einbezogen wurden, zeigt Tab. 2. Bei etwa der Hälfte, *n* = 53, bestand eine Medikation mit SSRI. Weiterhin häufig eingenommene AD gehören zu den Klassen der Serotonin-Noradrenalin-Wiederaufnahmehemmer (SSNRI), der noradrenerg/spezifisch serotonergen AD (NaSSA) und der selektiven Noradrenalin‑/Dopaminwiederaufnahmehemmer (SNDRI). Bei *n* = 7 der Patient*innen war der Gebrauch von Lorazepam während der Therapie bekannt und *n* = 14 nahmen während des Behandlungszeitraums Betablocker ein. Diese wurden nicht extra betrachtet und verteilen sich in etwa gleichmäßig auf die beiden Untersuchungsgruppen.

**Table 2:** 

Wirkstoffklasse	Präparat	Total	Dosis (mg/Tag)	
		*n*	M^b^	SD
SSRI	Citalopram	19	24,21	11,80
	Escitalopram	16	12,33	4,85
	Sertralin	12	97,92	45,80
	Paroxetin	4	27,50	5,00
	Fluoxetin	2	15,00	7,07
SNRI	Venlafaxin	16	11,19	59,49
	Duloxetin	3	50,00	17,32
NARI	Reboxetin	1	8,00	–
NaSSA	Mirtazapin	20	21,38	10,96
SNDRI	Opipramol	10	106,00	57,97
	Bupropion	1	150,00	–
TCA	Trimipramin	2	75,00	35,36
	Amitriptylin	2	50,00	35,36
	Doxepin	1	100	–
Andere	Agomelatin	2	25,00	0,00
	Vortioxetin	1	10,00	–
	Tianeptin	1	25,00	–

*M* Mittelwert, *SD* Standardabweichung, *SSRI* selektive Serotoninwiederaufnahmehemmer, *SNRI* Serotonin-Noradrenalin-Wiederaufnahmehemmer, *NARI* selektive Noradrenalinwiederaufnahmehemmer, *NaSSA* noradrenerg/spezifisch serotonerge Antidepressiva, *SNDRI* selektive Noradrenalin‑/Dopaminwiederaufnahmehemmer, *TCA* trizyklische Antidepressiva

^a^*n* = 13 Patient*innen nahmen 2 Präparate ein

^b^in Milligramm, *n* kann für manche Präparate aufgrund fehlender Werte abweichen

### Instrumente

Die Panik- und Agoraphobieskala (PAS; [3]) umfasst 13 Items und misst den Schweregrad einer Panik- bzw. agoraphobischen Symptomatik innerhalb der letzten Woche in den Bereichen: Panikattacken, agoraphobische Vermeidung, antizipatorische Angst, Einschränkung sowie Gesundheitsbefürchtungen. Die Items werden auf einer 5‑Punkte-Likert-Skala gemessen und der Gesamtwert kann Werte zwischen 0 und 52 annehmen. Die interne Konsistenz (Cronbachs α) liegt bei α = 0,86.

Die Revision des Beck-Depressionsinventars (BDI-II; [10]) ist ein Selbstbeurteilungsinstrument zur Erfassung des Schweregrads von Depression. Mittels 21 Fragen werden Existenz und Schweregrad depressiver Symptome nach DSM-IV beurteilt. Die Reliabilität des Messinstruments liegt im Bereich von α = 0,84 bis α = 0,94 (interne Konsistenz) in klinischen Stichproben.

### Statistische Analysen

Alle Analysen erfolgten mit SPSS Statistics, Version 29, IBM Corporation (Armonk, NY, USA). Zunächst wurden t‑Tests für unabhängige Stichproben oder χ^2^-Tests verwendet, um die Patient*innen mit gegenüber ohne AD hinsichtlich Panik- und agoraphober sowie depressiver Symptombelastung und deren Veränderung zu Therapieende, Diagnosenanzahl, Anteil komorbider depressiver Diagnosen zu vergleichen. Alle statistischen Tests waren zweiseitig. Das Signifikanzniveau wurde auf 0,05 angesetzt. Alphafehlerkorrekturen werden im Ergebnistext berichtet.

Anschließend wurden Responderanalysen mittels der Methode der prozentualen Symptomreduktion nach Hiller und Schindler [11] berechnet. Sie definieren Response durch zwei Kriterien. Zum einen als einen Punktwertrückgang von ≥ 50 % im pathologischen Bereich des Messinstruments, hier > 8, wie vorgeschlagen durch Bandelow [3], und zum anderen als eine Reduktion auf der Gesamtskala von ≥ 25 %. Patient*innen mit ≤ 8 Punkten zu Behandlungsende gelten als remittiert. Patient*innen, die keinen Punktwertrückgang von ≥ 50 % im pathologischen Bereich oder keinen Rückgang von ≥ 25 % auf der Gesamtskala verzeichnen, gelten als Nonresponder. Verläufe mit einer Zunahme von mehr als 25 % auf der Gesamtskala definierten wir als Verschlechterung. Patient*innen im nichtpathologischen Bereich zu Behandlungsbeginn werden extra ausgewiesen.

Schließlich wurde eine multiple lineare Regressionsanalyse mit der antidepressiven Medikation (kategorial: ja/nein) als unabhängige Variable und der Prä-post-Differenz des PAS-Gesamtwertes als abhängige Variable durchgeführt. Folgende Kontrollvariablen wurden in das Modell eingeschlossen: Störungsschwere zu Behandlungsbeginn (PAS-Gesamtwert zur Aufnahme); Depressivität (BDI-II-Gesamtwert zur Aufnahme), Alter und Geschlecht.

## Ergebnisse

Die Symptombelastung bei Therapieaufnahme und die Symptomveränderung bei PAS und BDI sowie Diagnosenanzahl und Anteil komorbider Depressionen verglichen zwischen Patient*innen mit und ohne bestehende Medikation mit AD zeigt Tab. 3. Mittels t‑Tests konnten keine signifikanten Unterschiede in der Panik- und agoraphoben Symptombelastung (*t *[333] = −1,712, *p* = 0,088, *d* = −0,21) sowie der depressiven Symptombelastung zu Beginn (*t *[93,753] = 0,047, *p* = 0,963, *d* = 0,01) festgestellt werden. In den PAS-Differenzen zeigten sich mittels t‑Test ebenfalls keine signifikanten Unterschiede (*t *[235] = −0,635, *p* = 0,526, *d* = −0,09). Es zeigte sich jedoch eine stärkere Verbesserung in Bezug auf Depressivität bei der Gruppe ohne Medikation. Dieser im Einzeltest signifikante Befund (*t *[295] = 2,196, *p* = 0,031, *d* = 0,40) Befund verliert jedoch nach Bonferroni-Korrektur des Alphafehlers seine statistische Signifikanz (*p* = 0,186 nach Multiplikation mit 6 Hypothesenprüfungen). In Bezug auf die Anzahl der gestellten Diagnosen (*t *[378] = −1,72, *p* = 0,087, *d* = −0,20) zeigte sich kein Unterschied. Allerdings erfüllten Patient*innen mit AD auch nach Bonferroni-Korrektur signifikant häufiger die Kriterien einer gegenwärtig komorbiden depressiven Störung (χ^2^ [1] = 18,321, *p* < 0,001, *φ* = 0,22).

**Table 3:** 

	Medikation				
	Ja		Nein		*p*
	*n*^a^ = 100		*n*^a^ = 280		
	*n*^*b*^	M (SD)	*n*^*b*^	M (SD)	
PAS_Anfang_	88	25,64 (9,80)	247	23,54 (9,93)	0,088^c^
PAS_Differenz_	65	9,56 (10,44)	172	8,69 (8,99)	0,526^c^
BDI-II_Anfang_	80	19,27 (15,30)	280	19,35 (8,62)	0,963^c^
BDI-II_Differenz_	66	5,26 (16,58)	231	9,98 (10,29)	0,031^c^
Anzahl Diagnosen	100	2,17 (0,92)	280	1,98 (0,94)	0,087^c^
Komorbid depressive Störungen, *n* (%)	100	58 (58,0 %)	280	94 (33,6 %)	< 0,001^d^

*M* Mittelwert, *SD* Standardabweichung, *PAS* Panik- und Agoraphobieskala, *BDI-II* Beck-Depressionsinventar II

^a^*n* Patient*innen der Gesamtstichprobe (*n* = 488) mit Daten über Antidepressivaeinnahme

^*b*^*n* Abweichungen durch Fragebogenmissings oder Nichtbeendigung der Therapie

^*c*^*p*-Werte t‑Tests vor Alphafehlerkorrektur

^d^*p*-Wert χ^2^-Tests vor Alphafehlerkorrektur

In Tab. 4 ist die Therapieresponseraten der Patient*innen in Bezug auf die Angstsymptomatik bei beiden Patient*innengruppen dargestellt, berechnet nach Hiller und Schindler [11], und somit die klinische Bedeutsamkeit der Veränderungen. Bei fast identischen Raten in beiden Gruppen zeigte ein χ^2^-Test keinen signifikanten Unterschied.

**Table 4:** 

	Antidepressiva	
Therapieresponse	Ja (*n*^b^ = 65)	Nein (*n*^b^ = 172)
	*n* (%)	*n* (%)
Remission	17 (26,2)	44 (25,6)
Response	18 (27,7)	48 (27,9)
Nonresponse	22 (33,8)	59 (34,3)
Verschlechterung	4 (6,2)	10 (5,8)
Zu Behandlungsbeginn in Remission	4 (6,2)	11 (6,4)

^a^Panik- und Agoraphobieskala

^b^*n* Abweichungen von *n* = 380 mit Daten zu Antidepressiva durch PAS-Missings

Die Assoziation einer bestehenden Medikation mit AD mit der PAS-Veränderung unter Berücksichtigung der Kontrollvariablen Störungsschwere, Depressivität, Alter und Geschlecht wurde mittels multipler linearer Regressionsanalyse untersucht (Tab. 5). Das Modell zeigt keine Aufklärung der Symptomverbesserung durch eine Medikation mit AD. Jedoch zeigte sich eine signifikante Assoziation der Störungsschwere zu Behandlungsbeginn (*t* = 8,17, *p* < 0,001) sowie des Alters (*t* = −2,03, *p* = 0,04) mit der PAS-Veränderung (*F *[5,219] = 16,52, *p* < 0,001, *n* = 225, *f*^*2*^ = 0,35). Anhand des Modells konnten 26 % der Varianz der Symptomveränderung zwischen Behandlungsbeginn und Behandlungsende erklärt werden.

**Table 5:** 

Einfluss auf den Therapieerfolg			
Prädiktoren	B	β	SE
*Antidepressiva vor der Therapie*	−0,53	−0,02	1,30
PAS_AN_	0,47***	0,49***	0,06
BDI-II_AN_	−0,03	−0,03	0,06
Alter	−0,10*	−1,20*	0,05
Geschlecht	−1,32	−0,07	1,15
Konstante	4,20	–	3,32
*Anpassungsgüteparameter*			
R^2^	0,27	–	–
Korr. R^2^	0,26	–	–
F (df = 5; 219)	16,52***	–	–

*n* = 225 aus *n* = 380 mit vorliegenden Daten zur Antidepressivaeinnahme, Abweichung durch Fragebogenmissings

*B* unstandardisierter Regressionskoeffizient, *β* standardisierter Regressionskoeffizient, *SE* Standardfehler, *AN* Anfang, *PAS* Panik- und Agoraphobieskala, *BDI-II* Beck-Depressionsinventar II

*p < 0,05

**p < 0,01

***p < 0,001

## Diskussion

Es wurde der Vorhersagewert einer bestehenden Medikation mit AD für die Veränderung einer Panik- und agoraphoben Symptomatik während einer hochstandardisierten intensiven KVT untersucht. Patient*innen mit AD profitierten hier entgegen unseren Erwartungen in gleichem Maße wie die ohne AD. Die Resultate sind so mit zwei vorherigen Arbeiten mit kleineren Stichproben in naturalistischen Settings [20, 25] vergleichbar und widersprechen den Befunden von Arch und Craske [2].

Dies kann als ein Hinweis für eine zumindest kurzfristige Überlegenheit einer Kombination aus AD und KVT in der Akutbehandlungsphase ohne Betrachtung längerfristiger Aspekte wie Absetzeffekte beim AD gegenüber einer Monotherapie gelten. Allerdings liegen keine Daten zur vorherigen Wirkung des AD allein vor. Viele Patient*innen mit AD in der vorliegenden Stichprobe könnten Nonresponder unter AD gewesen sein, während die Anzahl erfolgreich Remittierter unter AD allein hier nicht betrachtet werden kann. Zumindest sprechen die Ergebnisse für die Indikation für KVT bei Nichtansprechen oder starken Residuen der Angstsymptomatik unter AD.

Im Theorieteil beschriebene wirkungshemmende Effekte von AD auf KVT wie eine Glukokortikoidhemmung [22] oder eine Störung eines amygdalaabhängigen Lernens [7] erschienen hier als kaum wirksam. Möglicherweise wirkte die hier im Vergleich zu anderen naturalistischen Settings sehr hohe Expositionsfrequenz, die laut Szuhany et al. [26] lernhemmende Effekte von AD mindert, wozu im hier vorliegenden Design jedoch keine Aussagen ableitbar sind.

Ungünstige, die Motivation für Expositionstherapie hemmende Attribuierungseffekte von Fortschritten auf das AD scheinen hier ebenfalls kaum wirksam gewesen zu sein, möglicherweise auch wegen der annehmbar zeitlich weiter vorausgegangenen Ansetzung des AD, wodurch eine Attribution auf die KVT erleichtert sein könnte (temporale Kontiguität; [14]). Attributionseffekte könnten beim Absetzen der Medikation eine größere Rolle spielen als während der Akutbehandlungsphase [23]. Auch eine allgemeine Demoralisierung durch eine möglicherweise vorher bereits nur eingeschränkt wirksame andere Behandlung, hier durch AD, schien nicht wirksam.

In Bezug auf die hier gezeigte, entsprechend den Behandlungsempfehlungen für Depressionen [8] erwartbar häufiger vorgelegene komorbide Diagnose Depression der Gruppe mit AD zeigte sich allerdings nur eine vergleichbare depressive Symptombelastung zu Beginn wie bei denen ohne AD. Dies wäre durch eine bereits vorher erfolgte Wirkung des AD erklärbar. Die Gruppe mit AD zeigte weiterhin einen Trend eines geringeren Rückgangs bei der depressiven Symptombelastung während der KVT, mit ausgesprochen starker Streuung, annehmbar durch das hier häufigere Vorkommen depressiver Rezidive. In Bezug auf die Angstsymptomatik profitierte sie jedoch in gleichem Maße, obwohl depressive Komorbiditäten möglicherweise für eine komplexere Genese der Psychopathologie im Ganzen im Vergleich zu den Patient*innen mit geringerer Depressivität also „nur“ einer Angstsymptomatik sprechen.

Wie auch bei Nakano et al. [20] und Rufer et al. [25] stellte das Ausmaß der Panik- und agoraphobischen Symptombelastung zu Behandlungsbeginn einen bedeutsamen Prädiktor für das Therapieergebnis dar, möglicherweise aufgrund des statistischen Effekts einer Regression zur Mitte, aber auch durch ein günstigeres Ansprechen der auf die Angstsymptomatik fokussierende KVT bei ausgeprägterer Symptomatik. Der nichtsignifikante Vorhersagegehalt der weiteren Kontrollvariablen Geschlecht und Depressivität deckt sich ebenso mit den Ergebnissen vorausgegangener Arbeiten [20, 25]. Anders als bei vorliegenden Studien [20, 24] deuten die Ergebnisse hier an, dass ein höheres Alter zu Behandlungsbeginn mit einer geringeren Veränderung der Symptombelastung zwischen Aufnahme und Entlassung assoziiert sein könnte.

Zusammenfassend deuten unsere Ergebnisse darauf hin, dass eine bestehende antidepressive Medikation zumindest kurzfristig, und im Kontext einer hochfrequenten tagesklinischen auf Expositionen fokussierten KVT, die Erfolgsaussichten dieser nicht einzuschränken scheint.

Präparatspezifische Untersuchungen und Studien in ambulanten Settings bleiben notwendig, ebenso die Untersuchung der längerfristigen Verläufe, vor allem beim Absetzen von SSRI-Präparaten.

### Limitationen

Das naturalistische Forschungsdesign ist mit Einbußen der internen Validität verbunden, da zum einen keine Kontrolle der Interventionen und zum anderen keine randomisierten Kontrollgruppen vorlagen. Kausalschlüsse sind aufgrund des korrelativen Charakters der Arbeit unzulässig. Es lagen weiterhin keine Daten über Indikation, Zeitpunkt und Dauer der AD-Einnahme vor der Therapie vor, welche zur Einschätzung eines vorherigen alleinigen AD-Effekts von Bedeutung gewesen sein könnten. Es besteht zudem die Möglichkeit, dass weitere Prädiktoren, welche für die Vorhersage von Bedeutung waren, im multiplen linearen Regressionsmodell nicht berücksichtigt wurden. Um ein differenziertes Bild des Therapieerfolgs zu erhalten, wäre die Verwendung weiterer Instrumente zur Messung der Psychopathologie notwendig gewesen.

## Fazit für die Praxis

- Eine auf Expositionen fokussierte tagesklinische KVT zeigte, wie vorangegangene Studien [9, 15], hohe Response- und Remissionsraten bei der Behandlung von Panikstörung und/oder Agoraphobie.
- Patient*innen mit einer bestehenden antidepressiven Medikation unterschieden sich zu Behandlungsbeginn nicht signifikant in der Angst- und in der depressiven Symptombelastung von Patient*innen ohne antidepressive Medikation.
- Patient*innen mit einer bestehenden antidepressiven Medikation zu Behandlungsbeginn unterschieden sich nicht im Therapieerfolg in Bezug auf die Angstsymptomatik von Patient*innen ohne AD.
